# Administration of AMD3100 in endotoxemia is associated with pro-inflammatory, pro-oxidative, and pro-apoptotic effects in vivo

**DOI:** 10.1186/s12929-016-0286-8

**Published:** 2016-10-03

**Authors:** Semjon Seemann, Amelie Lupp

**Affiliations:** Institute of Pharmacology and Toxicology, Jena University Hospital, Friedrich Schiller University Jena, Drackendorfer Str. 1, 07747 Jena, Germany

**Keywords:** CXCR4, AMD3100, CXCL12, Endotoxemia, Oxidative stress

## Abstract

**Background:**

Chemokine receptor 4 (CXCR4) is a multifunctional G protein-coupled receptor that is activated by its natural ligand, C-X-C motif chemokine 12 (CXCL12). As a likely member of the lipopolysaccharide (LPS)-sensing complex, CXCR4 is involved in pro-inflammatory cytokine production and exhibits substantial chemo-attractive activity for various inflammatory cells. Here, we aimed to characterize the effects of CXCR4 blockade in systemic inflammation and to evaluate its impact on organ function. Furthermore, we investigated whether CXCR4 blockade exerts deleterious effects, thereby substantiating previous studies showing a beneficial outcome after treatment with CXCR4 agonists in endotoxemia.

**Methods:**

The CXCR4 antagonist AMD3100 was administered intraperitoneally to mice shortly after LPS treatment. After 24 h, health status was determined and serum tumor necrosis factor alpha (TNF alpha), interferon gamma (IFN gamma), and nitric oxide (NO) levels were measured. We further assessed oxidative stress in the brain, kidney, and liver as well as liver biotransformation capacity. Finally, we utilized immunohistochemistry and immunoblotting in liver and spleen tissue to determine cluster of differentiation 3 (CD3), CD8, CD68, and TNF alpha expression patterns, and to assess the presence of various markers for apoptosis and oxidative stress.

**Results:**

Mice treated with AMD3100 displayed impaired health status and showed enhanced serum levels of TNF alpha, IFN gamma and NO levels in endotoxemia. This compound also amplified LPS-induced oxidative stress in all tissues investigated and decreased liver biotransformation capacity in co-treated animals. Co-treatment with AMD3100 further inhibited expression of nuclear factor (erythroid-derived 2)-like 2 (Nrf-2), heme oxygenase-1 (HO-1), and various cytochrome P450 enzymes, whereas it enhanced expression of CD3, inducible nitric oxide synthase, and TNF alpha, as well as the total number of neutrophils in liver tissue. Spleens from co-treated animals contained large numbers of erythrocytes and neutrophils, but fewer CD3+ cells, and demonstrated increased apoptosis in the white pulp.

**Conclusions:**

AMD3100 administration in a mouse model of endotoxemia further impaired health status and liver function and mediated pro-inflammatory, pro-oxidative, and pro-apoptotic effects. This suggests that interruption of the CXCR4/CXCL12 axis is deleterious in acute inflammation and confirms previous findings showing beneficial effects of CXCR4 agonists in endotoxemia, thereby more clearly elucidating the role of CXCR4 in inflammation.

**Electronic supplementary material:**

The online version of this article (doi:10.1186/s12929-016-0286-8) contains supplementary material, which is available to authorized users.

## Background

Chemokine receptor 4 (CXCR4) is a multifunctional G protein-coupled receptor, activated by its natural ligand C-X-C motif chemokine 12 (CXCL12) as well as by macrophage migration inhibitory factor (MIF) and ubiquitin [1, 2]. Both CXCR4 and CXCL12 perform important biological functions during embryonic development and hematopoiesis and have pleiotropic roles in the immune system and during tissue repair processes [3]. The fundamental importance of CXCR4 has been demonstrated by the fact that mice lacking this receptor are unable to survive due to critical defects in leukocyte generation and hematopoiesis, leading to embryonic and neonatal fatalities, as well as defects in heart and brain development [4]. CXCL12 also exhibits substantial chemo-attractive activity for various cells, such as monocytes and T cells, both of which play critical roles in inflammatory processes [5, 6].

CXCR4 was previously found to be involved in the production of pro-inflammatory cytokines, such as interleukin 6 (IL-6), which is increased after CXCR4 activation in microglia, human oral cancer cells, and fibroblasts [7–9]. The authors attributed this observation to a substantial activation of phosphatidylinositol-4,5-bisphosphate 3-kinase (PI3K), nuclear factor ‘kappa-light-chain-enhancer’ of activated B-cells (NF-kB), and activator protein 1 (AP-1). Additionally, CXCR4 activation with CXCL12 increased TNF alpha mRNA and protein levels in primary astrocytes in vitro [10].

Despite these observations, the role of the CXCR4/CXCL12 axis in inflammatory diseases remains controversial and is not well characterized. Several authors have reported beneficial outcomes after treatment with various CXCR4 antagonists in models of rheumatoid arthritis, colitis, and lupus erythematodes [11–13]. Because elevated levels of CXCL12 were present in the affected tissue, blockade of CXCR4 resulted in a decreased infiltration with CXCR4+ cells, such as T cells and neutrophils, leading to a mitigation of the inflammatory conditions. In contrast, previous investigations from our group and others revealed a beneficial outcome after administration of a CXCR4 agonist in the lipopolysaccharides (LPS)-induced model of inflammation in vivo [14–16].

As a well-established animal model for systemic inflammation and septic shock, administration of LPS in mice can be used to study the anti-inflammatory potential of various drugs. LPS binds the lipopolysaccharide binding protein (LBP) and interacts with a receptor complex formed by CD14 (cluster of differentiation 14), MD-2 (myeloid differentiation protein-2), and toll-like receptor 4 (TLR4), which then activates TLR4-mediated signal transduction. This leads to increased NF-kB activation and enhanced production of proteases, reactive oxygen species (ROS), and nitrogen species (NOS) [17]. Pro-inflammatory cytokines are also produced, leading to an increased oxidative burst and decreased biotransformation capacity of the liver [18]. In regard to the numerous medications sepsis patients are usually treated with, the preservation of the biotransformation capacity is of substantial importance.

CXCR4 has been shown to be a component of the LPS-sensing complex, suggesting that treatment with CXCR4 agonists or antagonists could modulate TLR4 signaling [19]. However, little is known regarding the precise effects of CXCR4 blockade in endotoxemia. Therefore, in this study, we further aimed to unravel the systemic impact of such a blockade on LPS-induced organ damage, by treating mice with a combination of the CXCR4 antagonist AMD3100 and LPS. We hypothesized that several effects might only become visible by antagonizing the receptor, rather than administering a CXCR4 agonist, enabling us to understand the impact of CXCR4 in endotoxemia. We focused mainly on the health status of treated mice and specifically, whether a CXCR4 blockade would worsen endotoxemia, as suggested previously [14–16]. We further measured the effect of AMD3100 on production of pro-inflammatory cytokines, induction of oxidative stress in different tissues, and the liver biotransformation capacity. We focused on the liver and spleen as two crucial organs to determine the in vivo significance of the CXCR4/CXCL12 axis. Consequently, we intended to understand the impact of CXCR4 in endotoxemia more precisely and to explore its influence in inflammation from another perspective.

## Methods

### Animals and experimental procedure

The study was conducted under the license of the Thuringian Animal Protection Committee (approval number: 02–044/14). The principles of laboratory animal care and the German Law on the Protection of Animals, as well as the Directive 2010/63/EU were followed. Male adult C57BL/6 N mice (12-weeks-old, body weight 25–30 g; Charles River Laboratories, Sulzfeld, Germany) were used, and the animals were housed in plastic cages under standardized conditions (light-dark cycle 12/12 h, temperature 22 ± 2 °C, humidity 50 ± 10 %, pellet diet Altromin 1316, water ad libitum). A total of 30 mice were randomly divided into four groups: control, LPS, AMD3100 (*n* = 7 each), and AMD3100 plus LPS (*n* = 9). LPS (*Escherichia coli* 0111:B4, Sigma Aldrich, Steinheim, Germany) was injected intraperitoneally (5 mg/kg body weight, dissolved in phosphate-buffered saline [PBS]) and AMD3100 (5 mg/kg body weight, Tocris Bioscience, Bristol, UK) was administered in PBS intraperitoneally 2 h after endotoxemia onset. The most appropriate LPS dose, as well as the final time point, were determined in pilot studies, and the AMD3100 dose was selected based on previous publications [20, 21]. At 24 h post-LPS treatment, body temperatures were measured, and the condition of the animals was assessed using the Clinical Severity Score (CSS), as described previously [22]. Afterwards, the mice were sacrificed using isoflurane anesthesia, and their brains, kidneys, livers, and spleens were removed, weighed, and either fixed in 10 % buffered formaldehyde or snap-frozen in liquid nitrogen for biochemical analysis or immunoblotting, respectively. Additionally, whole blood was collected, and blood sugar levels were determined using a commercially available blood glucose meter and respective test strips (BG star®, Sanofi-Aventis, Frankfurt, Germany). Subsequently, serum was obtained and used for enzyme-linked immunosorbent assay (ELISA) and enzymatic activity measurements. For histological analysis, the formalin-fixed organ samples were embedded in paraffin blocks and cut into 4-μm thin sections (*n* = 7 for each treatment group).

### IFN gamma, TNF alpha, aspartate aminotransferase (ASAT), alanine aminotransferase (ALAT), nitric oxide (NO), urea, and creatinine assays

To determine the serum levels of IFN gamma, TNF alpha, ASAT, ALAT, and NO, a mouse IFN gamma ELISA kit (Pierce Biotechnology, Rockford, IL, USA), a mouse TNF-alpha Quantikine ELISA kit (R&D Systems, MA, USA), the EnzyChrom™ Aspartate Transaminase Assay Kit, the EnzyChrom™ Alanine Transaminase Assay Kit (both BioAssay Systems, Hayward, CA, USA) and the Nitrate/Nitrite Colorimetric Assay Kit (Cayman Chemical Company, Michigan, USA), respectively, were used according to the manufacturer instructions. Creatinine was determined by means of the Jaffé reaction. Briefly, in a strongly alkaline medium, picric acid is added to the sample. Under these conditions, it reacts with creatinine to form an orange-red complex, which can be measured photometrically at 492 nm. Serum urea was measured using the commercially available colorimetric Urea Assay Kit (Sigma-Aldrich Chemie GmbH, Steinheim, Germany), which utilizes coupled enzyme reactions involving urease and glutamate dehydrogenase, resulting in a product that can be detected at 570 nm.

### Oxidative status in the tissues

The tissue glutathione content in its reduced (GSH) and oxidized (GSSG) forms was analyzed by homogenizing the samples with 11 volumes of 0.2 M sodium phosphate buffer (5 mM ethylenediaminetetraacetic acid [EDTA]; pH 8.0) and four volumes of 25 % metaphosphoric acid. After centrifugation (12000 g, 4 °C, 30 min), GSH content was measured in the supernatants using a colorimetric assay, as previously described [23]. The GSSG concentration was assessed fluorometrically [24]. To determine the tissue content of lipid peroxides (LPO) as thiobarbituric acid-reactive substances (TBARS), liver samples were homogenized with 19 volumes of ice-cold saline and analyzed fluorometrically, as previously described [25].

### Biotransformation capacity

To obtain 9000 g supernatants for analysis, livers were homogenized with 0.1 M sodium phosphate buffer (pH 7.4) (1:2 w/v) and subsequently centrifuged at 9000 g for 20 min at 4 °C. The 9000 g supernatants were used to assess the activities of several cytochrome P450 (CYP) enzymes, and the protein content of these fractions was determined using a modified Biuret method [26]. For determination of CYP enzyme activities, the following model reactions were performed: ethoxycoumarin-O-deethylation (ECOD; [27]), ethoxyresorufin-O-deethylation (EROD; [28]), methoxyresorufin-O-demethylation (MROD; [28]), p-nitrophenol-hydroxylation (PNPH; [29]), and pentoxyresorufin-O-depentylation (PROD; [28]).

### Histopathology and immunohistochemistry

Samples for histopathology and immunohistochemistry were prepared by cutting 4-μm sections from the paraffin blocks and floating these onto positively charged slides. Immunostaining was performed by an indirect peroxidase-labeling method, as described previously [30]. Briefly, sections were de-waxed, microwaved in 10 mM citric acid (pH 6.0) for 16 min at 600 W, and incubated with the respective primary antibodies (Table 1) at 4 °C overnight. Detection of the primary antibody was performed using either a biotinylated goat anti-rabbit, a horse anti-mouse, or a rabbit anti-goat IgG, followed by incubation with peroxidase-conjugated avidin (Vector ABC “Elite” kit, Vector, Burlingame, CA, USA). Binding of the primary antibody was visualized using 3-amino-9-ethylcarbazole (AEC) in acetate buffer (BioGenex, San Ramon, CA, USA). The sections were then rinsed, counterstained with Mayer’s hematoxylin (Sigma Aldrich, Steinheim, Germany), and mounted in Vectamount™ mounting medium (Vector Laboratories, Burlingame, CA, USA). Additionally, TUNEL (TdT-mediated dUTP-biotin nick end labeling) staining was performed using the In Situ Cell Death Detection Kit, POD (Roche Diagnostics, Mannheim, Germany), according to the manufacturer instructions. All immunohistochemical stainings were evaluated by two independent investigators. To detect the liver glycogen content, periodic-acid-Schiff staining (PAS; periodic acid, Schiff’s reagent: Sigma Aldrich, Steinheim, Germany) was performed, using standard protocols [31]. Identification of the specific cell types was based on their microscopic features along with the relative location of the cells in the respective tissues.

**Table 1:** Primary antibodies used for immunohistochemistry (IHC) and immunoblotting (IB)

Primary antibody	Type, Catalogue number	Manufacturer	Dilution IHC/IB	Host species
CD3	polyclonal, sc-20047	Santa Cruz Biotechnology	1:500/1:200	Mouse
CD8	polyclonal, sc-7188	Santa Cruz Biotechnology	1:200/-	Rabbit
CD68/ED1	monoclonal, MCA341R	AbDSerotec	1:50/-	Mouse
cleaved caspase-3	monoclonal, 9661	Cell Signaling Technology	1:600/1:1000	Rabbit
CYP3A	polyclonal	Daiichi Pure Chemicals	1:5000/1:10000	Goat
CYP2B	polyclonal	Daiichi Pure Chemicals	1:5000/1:10000	Goat
CYP2E1	polyclonal	Daiichi Pure Chemicals	1:5000/1:10000	Goat
heme oxygenase 1	polyclonal, SPA-895	Biomol GmbH	1:5000/1:10000	Rabbit
iNOS	polyclonal, sc-651	Santa Cruz Biotechnology	1:500/-	Rabbit
Nrf-2	polyclonal, sc-722	Santa Cruz Biotechnology	-/1:500	Rabbit
TNF alpha	monoclonal, sc-52746	Santa Cruz Biotechnology	1:500/-	Mouse
VEGF	monoclonal, sc-7269	Santa Cruz Biotechnology	1:500/-	Mouse

### Immunoblotting

Frozen liver and spleen samples (*n* = 4 from all treatment groups) were weighed and added (1:4) to detergent buffer (50 mM Tris-HCl, pH = 7.4, 150 mM NaCl, 5 mM EDTA, 10 mM NaF, 10 mM disodium pyrophosphate, 1 % Nonidet P-40, 0.5 % sodium deoxycholate, 0.1 % sodium dodecyl sulfate [SDS]) in the presence of protease and phosphatase inhibitors (Complete Mini and PhosSTOP; Roche Diagnostics, Mannheim, Germany). The samples were then sonicated for 10 s and gently inverted for 1 h at 4 °C before centrifugation for 30 min at 14800 g at 4 °C. Next, samples were diluted with SDS sample buffer (62.5 mM Tris-HCl, pH = 7.6, 2 % SDS, 20 % glycerol, 100 mM dithiothreitol, 0.005 % bromophenol blue), heated to 95 °C for 10 min, cooled to room temperature, and subsequently subjected to 10 % SDS-polyacrylamide gel electrophoresis (SDS-PAGE) and blotted onto polyvinylidene fluoride (PVDF) membranes. Liver blots were incubated with anti-CYP3A2, anti-CYP2B1, anti-CYP2E1, or anti-heme oxygenase-1 antibodies, whereas spleen blots were incubated with anti-cleaved caspase-3, anti-Nrf-2, or anti-CD3 antibodies, followed by incubation with peroxidase-conjugated anti-rabbit or anti-mouse secondary antibodies (Santa Cruz Biotechnology, Heidelberg, Germany; dilution 1:5000) and enhanced chemiluminescence detection (Thermo Scientific, Rockford, USA). β-actin, used as a loading control, was detected using a monoclonal mouse antibody (sc-47778, Santa Cruz Biotechnology, Heidelberg, Germany). All experiments were performed in quadruplicate.

### Blood cell quantification in the peripheral blood and in liver and spleen

At 24 h post-LPS treatment, blood was collected from all mice and transferred to vials containing EDTA in order to prevent clotting. The samples were then analyzed using a Sysmex pocH-100iV Diff hematology analyzer. Additionally, iNOS-positive neutrophils in the livers and in the spleens of all mice were counted in 10 independent visual fields each at a magnification of 630× or 200×, respectively, using a light microscope.

### Statistical analysis

All statistical analyses and figures were computed with GraphPad Prism software, v. 6.0 (GraphPad Software, La Jolla, CA, USA). In all cases, experiments were performed with seven animals per experimental group, except for the immunoblots, which were carried out in duplicate, with four animals per experimental group. Statistical significance was determined by using the one-way analysis of variance (ANOVA) and the Tukey post-hoc test, except for the CSS and the different blood cell types, which were analyzed by the non-parametric Kruskal-Wallis test, followed by the Mann-Whitney *U* test. A *p* value <0.05 (*) was considered as statistically significant; a *p* value <0.01 (**) and a *p* value <0.001 (***) are further specified. Data are presented as mean ± standard error of the mean (SEM), except for CSS and for the quantification of the different blood cell types, which are presented as medians, with interquartile ranges.

## Results

### Mortality, health status, weight development, and body temperatures

To assess the effect of CXCR4 blockade on LPS-mediated injury, male adult C57BL/6 N mice were treated intraperitoneally with LPS, AMD3100, AMD3100 plus LPS, or PBS (control) (*n* = 7 for all groups except AMD3100 plus LPS, where *n* = 9). We first conducted preliminary investigations to confirm the appropriate LPS dose and found that 5 mg/kg body weight was suitable in terms of causing no mortality within 24 h. However, in our LPS plus AMD3100 group, two out of the nine mice died, and these animals were not used for further analysis.

**Fig. 1:**
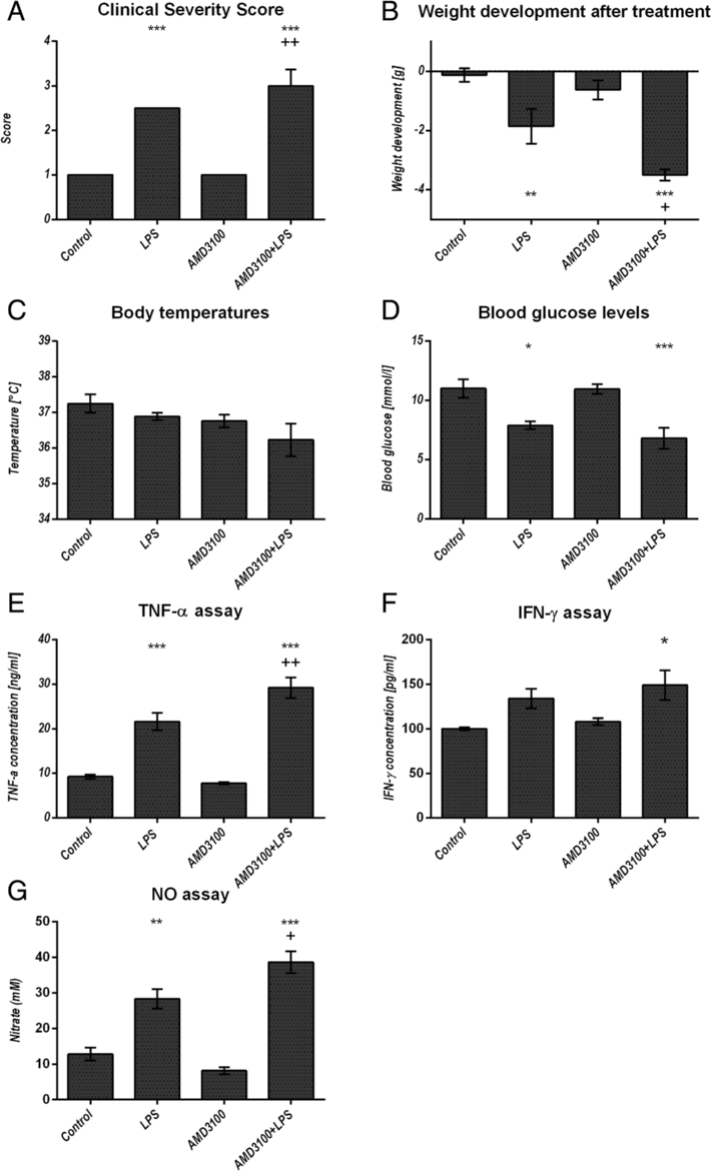
General condition and systemic parameters. C57BL/6 N mice were treated either with LPS (5 mg/kg body weight), AMD3100 (5 mg/kg body weight), with both substances or with the solvent PBS (control). 24 h after LPS administration, the Clinical Severity Score (**a**), the body weight (**b**) and the body temperature (**c**) were assessed and the mice were sacrificed. Blood glucose content was determined from whole blood (**d**) and serum was obtained for TNF alpha, interferon (IFN) gamma and nitric oxide (NO) measurements (**e**-**g**). Data are given as mean ± standard error of the mean (SEM) or as median with interquartile ranges (CSS), respectively; n = 7 for each group. Statistical significant differences between the different treatment groups were determined by using the one-way analysis of variance (ANOVA) and the Tukey post hoc test, except for the CSS, which was analyzed by the non-parametric Kruskal-Wallis test followed by the Mann-Whitney-*U* test. They are indicated as follows: *, *p* < 0.05; **, *p* < 0.01; ***, *p* < 0.001 vs. control animals; ^+^, *p* < 0.05; ^++^, *p* < 0.01; ^+++^, *p* < 0.001 vs. LPS treatment

The remaining animals were evaluated 24 h after LPS treatment, and we found that those receiving LPS displayed an impaired health status as compared to controls, which was even more severe after co-administration of AMD3100 and LPS, as evidenced by increased CSSs, in comparison to the control and LPS-treated mice (Fig. 1a). Specifically, co-treated mice showed less activity, moved notably slower and with more difficulty, slept more often, and exhibited a ruffled fur, as compared to the other groups of animals. Further, these mice consumed less food and water than even the mice treated with LPS alone, which, *inter alia*, led to a weight loss (Fig. 1b). Treatment of the animals with LPS alone or with AMD3100 alone led to reduced body temperatures when compared with the control mice. In accordance with the other data, an additive effect was observed in animals receiving both AMD3100 and LPS (Fig. 1c).

### Blood count, blood glucose, serum TNF alpha, IFN gamma, NO, creatinine, and urea levels

**Fig. 2:**
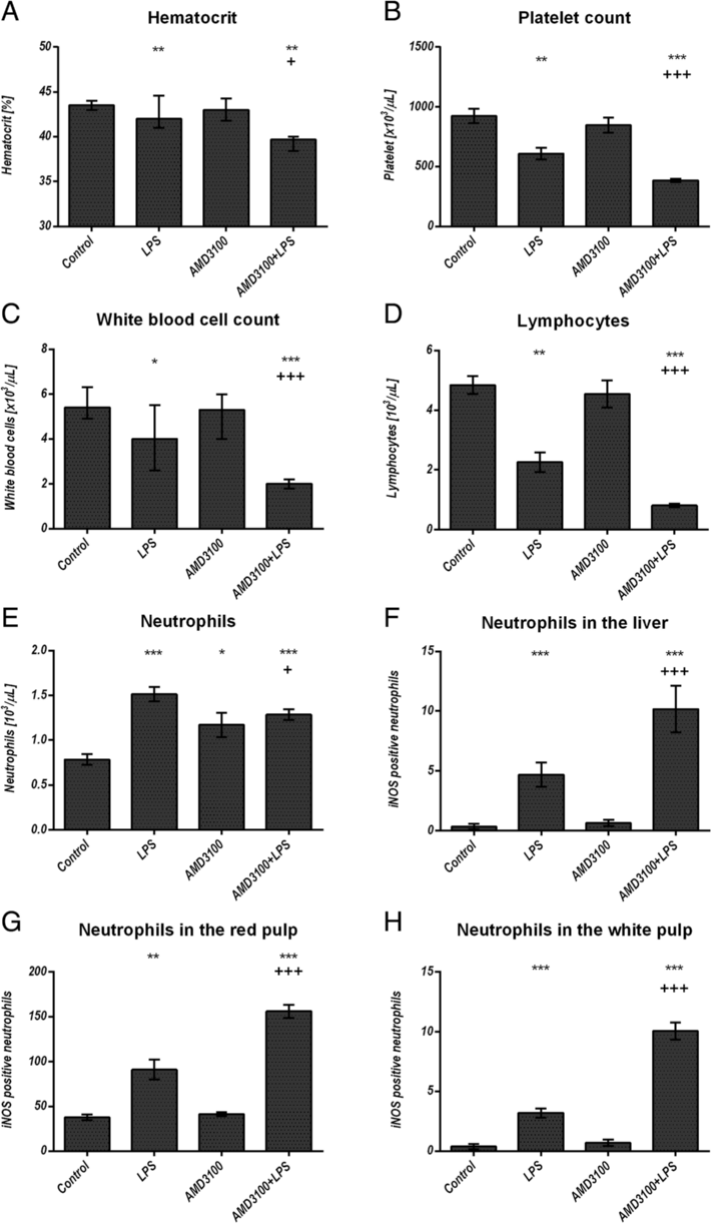
Blood cell quantification in the peripheral blood and in the organs. C57BL/6 N mice were treated either with LPS (5 mg/kg body weight), AMD3100 (5 mg/kg body weight), with both substances or with the solvent PBS (control). 24 h after LPS administration, the mice were sacrificed and the blood as well as the livers and spleens were collected. 50 μl of each blood sample were analyzed by using the Sysmex pocH-100iV Diff hematology analyzer for hematocrit, platelet, white blood cell, lymphocyte and neutrophil count (**a**-**e**). Liver (**f**) and spleen sections (**g**-**h**) of each mouse were stained by means of immunohistochemistry for iNOS expression and iNOS positive neutrophils were counted in ten independent visual fields each at a magnification of 630× or 200×, respectively, using a microscope. Data are given as median with interquartile ranges; n = 7 for each group. Statistical significant differences between the different treatment groups were determined by using the non-parametric Kruskal–Wallis followed by the Mann-Whitney-*U* test and indicated as follows: *, *p* < 0.05; **, *p* < 0.01; ***, *p* < 0.001 vs. control animals; ^+^, *p* < 0.05; ^++^, *p* < 0.01; ^+++^, *p* < 0.001 vs. LPS treatment

Administration of LPS led to a decreased hematocrit and reduced the amount of platelets and white blood cells, when compared to the control group. However, the additional AMD3100-mediated CXCR4 blockade reduced all these parameters even further. In contrast, the neutrophil count in the peripheral blood was enhanced after endotoxin challenge, whereas animals co-treated with AMD3100 and LPS contained slightly fewer circulating cells in comparison to the LPS group. Additionally, AMD3100 alone was able to cause neutrophilia, when compared to the PBS treatment control (Fig. 2a-e). To further determine the systemic effect of a CXCR4 blockade, we assessed the amount of glucose in whole blood, as well as the levels pro-inflammatory cytokines and NO in the serum. After 24 h, endotoxin treatment induced hypoglycemia, whereas co-administration of AMD3100 and LPS reduced blood glucose levels even further (Fig. 1d). Moreover, administration of LPS triggered an elevation of serum TNF alpha and IFN gamma levels by about 250 and 30 %, respectively, as compared to the control group. AMD3100 in conjunction with LPS further increased the serum levels of both cytokines in endotoxic mice by more than 35 and 12 %, respectively (Fig. 1e and f). LPS challenge also induced higher serum NO levels, indicating increased oxidative stress, and CXCR4 blockade further amplified these effects, thereby leading to the highest NO levels observed (Fig. 1g). Finally, blocking CXCR4 in endotoxemia produced higher serum creatinine levels, as compared to the control or to the LPS groups. In contrast, serum urea levels were decreased after LPS and (even more distinctly) after AMD3100 plus LPS challenge (Additional file 1 b, c). For all parameters investigated, other than neutrophil count, no relevant influence of AMD3100 alone was detectable.

### Oxidative stress in different tissues

**Fig. 3:**
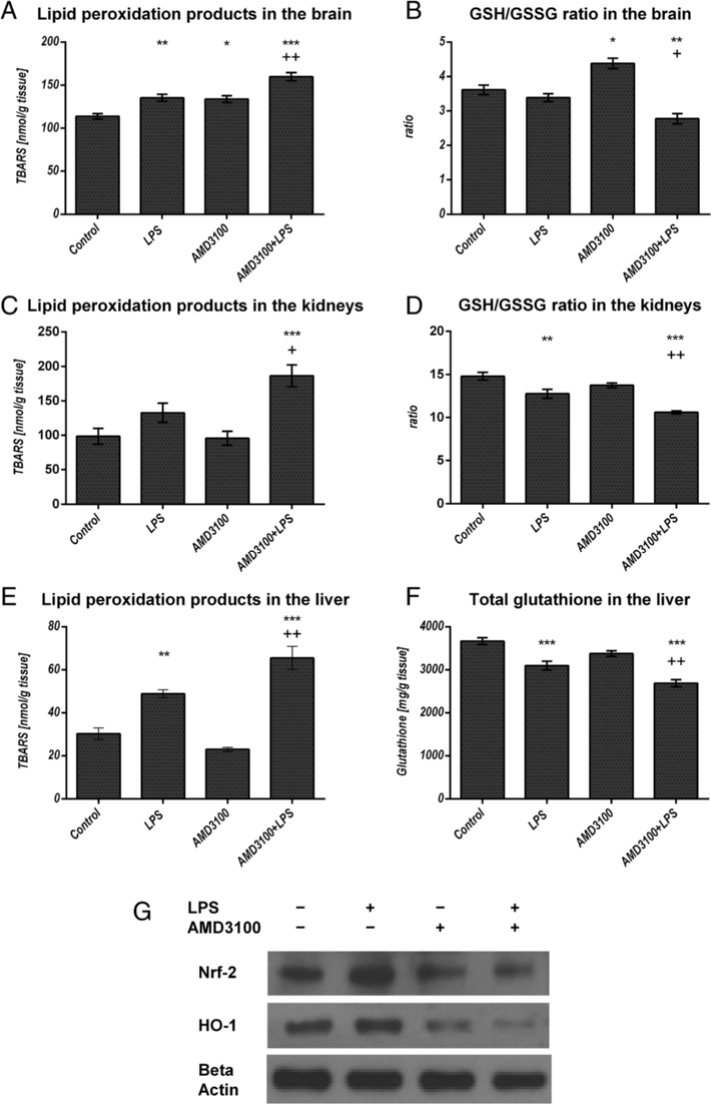
Oxidative stress in different organs. C57BL/6 N mice were treated either with LPS (5 mg/kg body weight), AMD3100 (5 mg/kg body weight), with both substances or with the solvent PBS (control). 24 h after LPS administration, the mice were sacrificed and different organs were collected for the analysis of the tissue content of lipid peroxidation products as determined by thiobarbituric acid reactive substances (TBARS) (**a**, **c**, **e**). As additional parameters, the GSH/GSSG ratio in the brain and kidneys (**b**, **d**) and the total glutathione content in the liver (**f**) are depicted. Data are given as mean ± standard error of the mean (SEM), n = 7 for each group. Statistical significant differences between the different treatment groups were determined by using the one-way analysis of variance (ANOVA) and the Tukey post hoc test and are indicated as follows: *, *p* < 0.05; **, *p* < 0.01; ***, *p* < 0.001 vs. control animals; ^+^, *p* < 0.05; ^++^, *p* < 0.01; ^+++^, *p* < 0.001 vs. LPS treatment. In (**g**) representative examples of n = 4 independent immunoblot analyses of Nrf-2 and HO-1 expression in the liver are shown

Due to the increased serum NO levels in mice treated with LPS and LPS plus AMD3100, we assessed the oxidative status in different organs. Therefore, we quantified the lipid peroxidation products (LPO), as well as the levels of reduced (GSH) and oxidized glutathione (GSSG) in the brains, kidneys, and livers of treated and control mice. We found that 24 h after endotoxemia onset, increased oxidative stress was detectable in all organs investigated. In the brains, LPS induced an elevated production of LPO, while co-administration of AMD3100 and LPS produced even higher levels (Fig. 3a). In parallel, the GSH/GSSG ratio was decreased due to a reduced amount of GSH (Fig. 3b). Interestingly, AMD3100 treatment alone also increased LPO production and produced an enlarged GSH/GSSG ratio. The oxidative states in the kidneys and in the livers were found to be very similar (Fig. 3c-f). These results demonstrate that endotoxin induces ROS production, which is indirectly measureable by the increased LPO content and the impaired glutathione status, and critically, co-treatment with AMD3100 worsens these effects even further. The liver, in particular, was strongly affected, as this organ showed approximately 35 % higher LPO values and 15 % less total glutathione content in animals co-treated with LPS and AMD3100, as compared to the LPS group.

### Liver

**Fig. 4:**
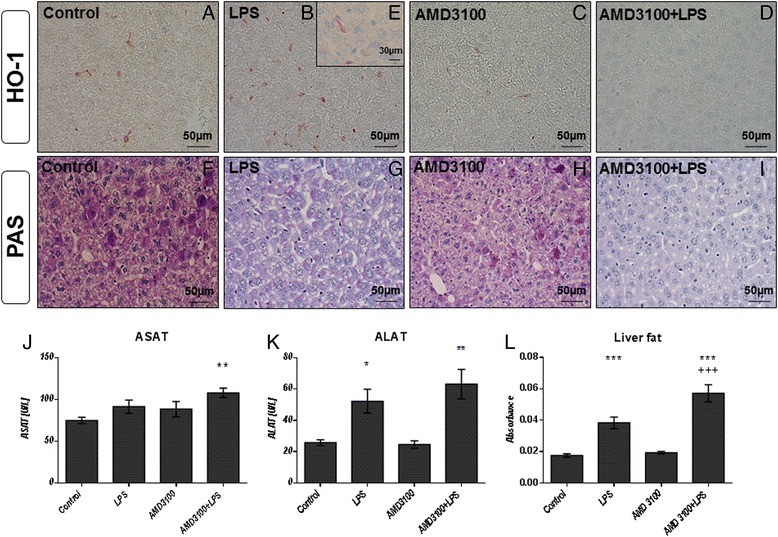
Heme oxygenase-1 expression, serum ASAT, ALAT levels, glycogen and total fat content in the livers. C57BL/6 N mice were treated either with LPS (5 mg/kg body weight), AMD3100 (5 mg/kg body weight), with both substances or with the solvent PBS (control). 24 h after LPS administration, the mice were sacrificed and blood and livers were collected for further biochemical and histological analysis. (**a**-**i**): Representative photomicrographs from one of seven different tissue samples stained for HO-1 expression are shown (immunohistochemistry (red-brown color), counterstaining with hematoxylin, original magnification: (**a**-**d**, **f**-**i**) 400×, (**c**) 630×). The photomicrograph in (**e**) shows a single Kupffer cell at a higher magnification (630×). As an approximate measurement for the fat content, we determined the turbidity value in the 9000 g supernatants of the livers (**l**). **j** and **k** depict the serum concentrations of ASAT and ALAT of all treatment groups. Data are given as mean ± standard error of the mean (SEM); *n* = 7 for each group. Statistical significant differences between the different treatment groups were determined by using the one-way analysis of variance (ANOVA) and the Tukey post hoc test and are indicated as follows: *, *p* < 0.05; **, *p* < 0.01; ***, *p* < 0.001 vs. control animals; ^+^, *p* < 0.05; ^++^, *p* < 0.01; ^+++^, *p* < 0.001 vs. LPS treatment; #, *p* < 0.05; ##, *p* < 0.01; ###, *p* < 0.001 vs. AMD3100 administration

To better understand the underlying cause(s) of oxidative stress observed in the different organs investigated, we used immunoblotting and immunohistochemistry. Here, we focused on the anti-oxidative enzymes, HO-1 and Nrf-2. As shown in Fig. 3g, our immunoblots revealed that HO-1 was induced after LPS challenge. In contrast, AMD3100 treatment alone led to a decrease in HO-1 expression, and after co-administration of AMD3100 and LPS, a further reduction was observed. These data were confirmed by our immunohistochemical analysis (Fig. 4a-e). Here, HO-1 expression could be detected mainly in Kupffer and in some pit cells, an effect that was clearly visible after LPS challenge. Again, we see that AMD3100 alone caused a distinct decrease in HO-1 expression in comparison to both LPS-treated and control animals, and after co-administration of AMD3100 and LPS, HO-1 expression was almost completely abolished. For Nrf-2, immunoblot analysis revealed an up-regulation after LPS administration, whereas co-treatment with AMD3100 and endotoxin led to a decreased Nrf-2 expression in the tissue (Fig. 3g).

To further determine how CXCR4 blockade could influence liver integrity, we measured the serum levels of ALAT and ASAT, ALAT, however, representing a more specific indicator of liver inflammation. In comparison to the control, LPS challenge caused an increase in ASAT values by about 20 %. However, AMD3100 alone was also able to induce ASAT levels to a comparable extent, and an additive effect was observed after combined treatment with AMD3100 and LPS (Fig. 4j). Similarly, endotoxin treatment augmented the serum concentrations of ALAT by about 100 % in comparison to the control, and the additional CXCR4 blockade caused a further increase in ALAT levels by about 20 % (Fig. 4k), indicating severe hepatocellular damage.

In light of these results and in order to gain a more detailed understanding of liver function in treated animals, we first performed periodic acid-Schiff staining as a measure for liver glycogen content (Fig. 4e-h). Exposure to LPS produced a massive loss of glycogen in the livers, although some glycogen reserves were still detectable. However, the additional CXCR4 blockade was able to remove all glycogen reserves from hepatocytes, and the livers from co-treated mice showed structural changes, which could be attributed to slight edema and intense fat accumulation. When determining the liver protein content as a reference for the CYP model reactions, we additionally assessed the turbidity value of each sample and used this as an approximation of the liver fat content. In concordance with our histological data, AMD3100 caused an additional fat accumulation in endotoxin-treated mice (Fig. 4l). While the samples of LPS treated mice were more turbid than the controls by about 110 %, the additional CXCR4 blockade provoked an additional 220 % increase. In parallel, a significant loss in liver protein content was observed. Whereas LPS provoked a decrease by about 9 %, co-treatment caused a >15 % loss, when compared with the control mice receiving PBS only (see Additional file 1 a).

**Fig. 5:**
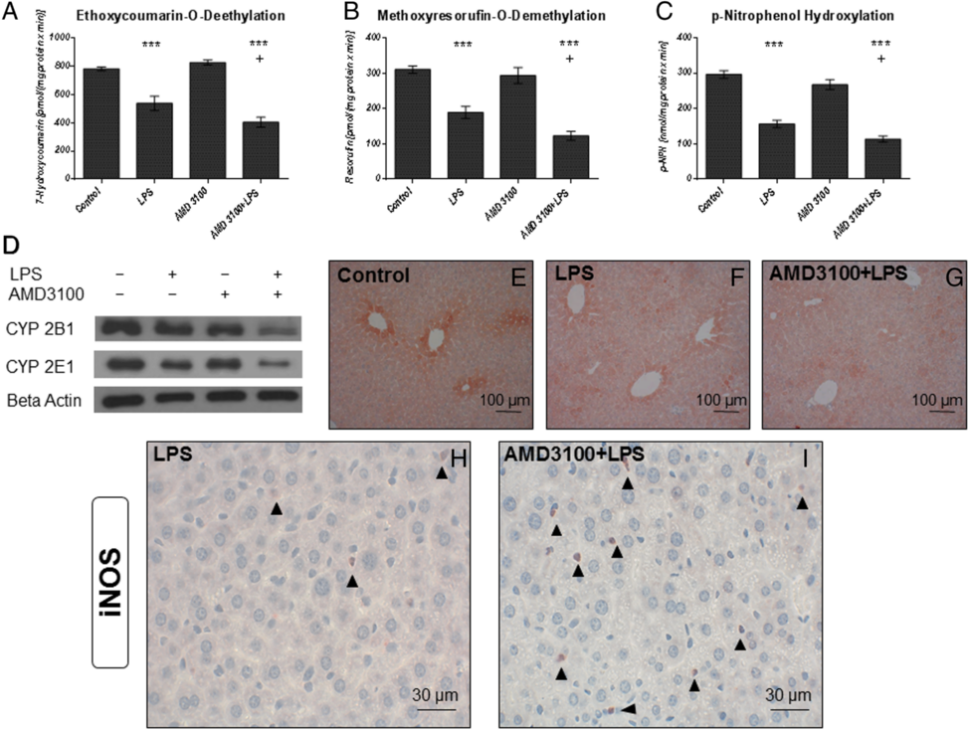
Biotransformation capacity and iNOS expression in the livers. C57BL/6 N mice were treated either with LPS (5 mg/kg body weight), AMD3100 (5 mg/kg body weight), with both substances or with the solvent PBS (control). 24 h thereafter, the mice were sacrificed and the livers were collected for biochemical and histological analysis. Ethoxycoumarin-O-deethylation [ECOD] (**a**), methoxyresorufin-O-demethylation [MROD] (**b**) and p-nitrophenol-hydroxylation [PNPH] (**c**) activities in 9000 g supernatants are shown exemplarily. Data are given as mean ± standard error of the mean (SEM); n = 7 for each group. Statistical significant differences between the different treatment groups were determined by using the one-way analysis of variance (ANOVA) and the Tukey post hoc test and are indicated as follows: *, *p* < 0.05; **, *p* < 0.01; ***, *p* < 0.001 vs. control animals; ^+^, *p* < 0.05; ^++^, *p* < 0.01; ^+++^, *p* < 0.001 vs. LPS treatment. **d**: CYP2B and CYP2E1 expression in liver tissue as determined by immunoblotting. Representative examples of n = 4 independent immunoblots are depicted. **e** and **g**: CYP 2B isoforms expression in liver tissue as determined by immunohistochemistry (red-brown color, counterstaining with hematoxylin). Representative photomicrographs from one of seven different liver tissue samples are shown (original magnification: 200×). **h** and **i**: iNOS expression in liver tissue as determined by immunohistochemistry (red-brown color, counterstaining with hematoxylin). Representative photomicrographs from one of seven different liver tissue samples are shown (magnification: 630×, arrowheads: iNOS expressing infiltrating neutrophil granulocytes)

Another crucial parameter for assessing liver function is the biotransformation capacity. As expected from our previous investigations on the effects of CTCE0214D in endotoxemia, we found that LPS caused a distinct loss in the activity of several CYP enzymes in the liver. Notably, whereas endotoxin decreased the activities of CYP1A, 2A, 2B, and 2C (ECOD) to approximately 70 % of the control values, the co-treatment further reduced all activities by about 25 % (Fig. 5a). Similar results were obtained when measuring the activities of CYP1A (EROD), CYP1A2 (MROD), CYP2B (PROD), and CYP1E (PNPH) (exemplary depicted in Fig. 5b and c). In comparison to LPS alone, additional administration of the CXCR4 antagonist further diminished CYP activities by approximately 30, 40, 25, and 30 %, respectively, when compared to the LPS group. Similar results were obtained in the corresponding Western blot analyses. Here, our data indicate that endotoxin treatment reduced expression of CYP2B, CYP2E1, and CYP3A in liver tissue (Fig. 5d), and the addition of AMD3100 led to a further decline in CYP enzyme expression; CYP2B isoforms, in particular, were strongly affected. These results were further confirmed by our immunohistochemical findings. As shown in Fig. 5e, the CYP enzymes are predominantly expressed around the central veins. However, 24 h after LPS challenge, CYP expression was notably decreased, and after co-administration of AMD3100, a further decrease was observed (Fig. 5f and g).

**Fig. 6:**
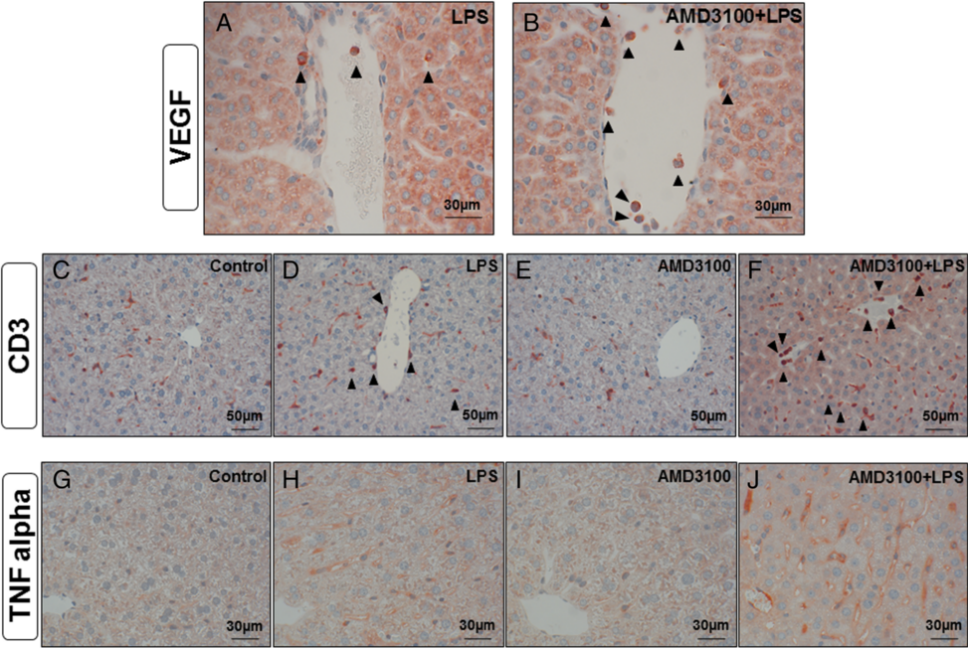
Immunohistochemical evaluation of VEGF, CD3 and TNF alpha expression. C57BL/6 N mice were treated either with LPS (5 mg/kg body weight), AMD3100 (5 mg/kg body weight), with both substances or with the solvent PBS (control). 24 h thereafter, the mice were sacrificed and the livers were collected for immunohistochemical analysis of VEGF, CD3 and TNF alpha expression (red-brown color, counterstaining with hematoxylin). Representative photomicrographs from one of seven different tissue samples each are shown (original magnification: (**a**, **b**) 630×, (**c**-**f**) 400×, (**g**-**j**) 630×)

In order to further clarify the mechanisms underlying these effects, we performed additional immunohistochemical stainings. Specifically, in accordance with the massive oxidative stress observed in the livers of mice treated with AMD3100 plus LPS, iNOS stainings revealed several iNOS-overexpressing neutrophil granulocytes that had apparently entered the tissue. This effect distinctly exceeded that seen after treatment with LPS alone (Fig. 2f and 5h, i). Similar results were observed in the corresponding staining for vascular endothelial growth factor (VEGF). While some VEGF-positive granulocytes were seen in the periportal regions of mice that had received LPS alone (Fig. 6a), after co-treatment with AMD3100 and endotoxin, a larger number of granulocytes were observed infiltrating the liver tissue and spreading throughout the liver lobules (Fig. 6b).

To approximate the amount of T cells in the tissue, we used CD3 as a marker because of its presence at all stages of T-cell development (Fig. 6c-f). While some CD3+ Kupffer and pit cells were scattered throughout the liver lobules in all treatment groups, after LPS treatment, additional CD3+ lymphocytes and granulocytes were detectable in the periportal regions, some of which had already entered the tissue (Fig. 6d). Again, in comparison to the LPS group, CXCR4 blockade with AMD3100 further enhanced the amount of CD3+ cells present throughout the liver lobules. Figure 6f shows several CD3+ granulocytes and lymphocytes infiltrating the liver after co-treatment with LPS and AMD3100.

We then aimed to determine the liver cell population responsible for the production of the elevated TNF alpha serum levels observed in endotoxemia, particularly after co-treatment with AMD3100 and LPS. Therefore, we performed TNF alpha stainings in the liver tissue from all treatment groups (Fig. 6g-j). As clearly evident from Fig. 6j, sinusoidal endothelial cells are the major source of TNF alpha production. Besides some Kupffer and pit cells, these endothelial cells very strongly express this pro-inflammatory cytokine when the livers are co-challenged with AMD3100 and LPS for 24 h. Though to a much lesser extent, endotoxin itself was also able to induce TNF alpha expression, whereas the livers of control and AMD3100-treated animals exhibit only few TNF alpha-positive cells.

### Spleen

**Fig. 7:**
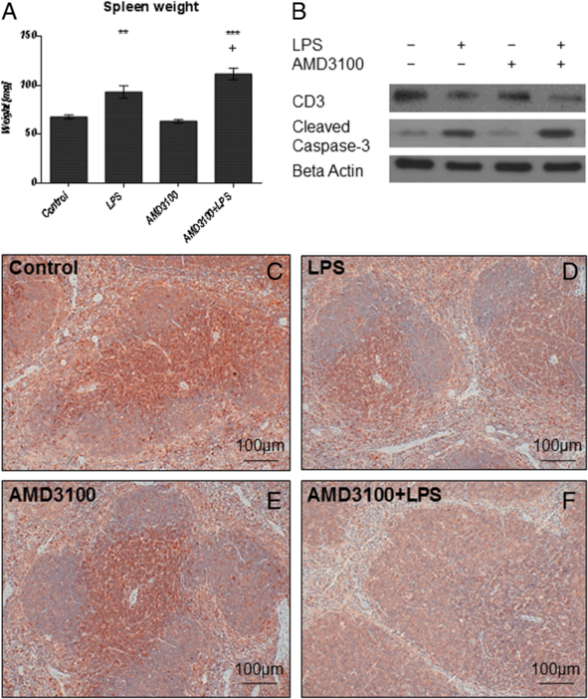
Spleen weights and CD3 and cleaved caspase-3 expression in the spleen. C57BL/6 N mice were treated either with LPS (5 mg/kg body weight), AMD3100 (5 mg/kg body weight), with both substances or with the solvent PBS (control). 24 h thereafter, the mice were sacrificed and the spleens were collected for further analysis. **a**: Spleen weights. Data are given as mean ± standard error of the mean (SEM); *n* = 7 for each group. Statistical significant differences between the different treatment groups were determined by using the one-way analysis of variance (ANOVA) and the Tukey post hoc test and are indicated as follows: *, *p* < 0.05; **, *p* < 0.01; ***, *p* < 0.001; ^+^, *p* < 0.05; ^++^, *p* < 0.01; ^+++^, *p* < 0.001 vs. LPS. **b**: CD3 and cleaved caspase-3 expression in the spleen as determined by immunoblotting. Representative immunoblots of *n* = 4 independent analyses are shown. **c**-**f**: cleaved caspase-3 expression in the spleen as determined by immunohistochemistry (red-brown color, counterstaining with hematoxylin). Representative photomicrographs from one of seven different tissue samples each are shown (original magnification: 200×)

The spleen is crucial for removing damaged red blood cells and bacteria from the bloodstream. Therefore, we were interested in how this organ responds to LPS, as well as to the AMD3100 plus LPS challenge. We found that LPS causes splenomegaly, as the spleen weights were significantly increased by about 40 % after 24 h of treatment, in comparison to those from the control group (Fig. 7a). Histological examination of the spleen further revealed an increased number of erythrocytes in the red pulp and a modest accumulation of neutrophils, along with a mild edema in the white pulp after LPS treatment. Critically, administration of the CXCR4 blockade with AMD3100 intensified all these effects, and 24 h post-treatment, the spleen weights from these animals were about 20 % higher than those from mice treated with LPS alone.

We next sought to determine the distribution of CD3+ cells in the spleen, in order to determine how these results compare to our data from the liver. We observed a large number of T-lymphocytes surrounding the periarteriolar lymphoid sheaths in the white pulp of spleens from control animals, and from mice treated with AMD3100 alone (Figs. 7c and e). In comparison, endotoxin challenge led to a massive efflux of CD3+ cells from the white pulp (Fig. 7d). Again, this effect was much more pronounced after CXCR4 blockade with AMD3100, and here, nearly no CD3+ lymphocytes were detectable in the spleen tissue (Fig. 7f). In combination with our findings from the liver, these results indicate that under inflammatory conditions, CD3+ cells seem to leave the spleen and to migrate to other organs, such as the liver.

To further substantiate our immunohistochemical data, we then performed additional immunoblotting experiments. As shown in Fig. 7b, administration of LPS caused a distinct reduction in CD3 expression in the spleen tissue, as compared to the control and to the AMD3100-only treatment. This effect was more pronounced after additional CXCR4 blockade with AMD3100. In parallel, we also examined the presence of CD8+ and CD68+ cells in the spleen tissue by immunohistochemistry. Here, in comparison to the control and to the AMD3100-only treatment, a clear increase in the number of CD8+ and CD68+ cells could be seen in the white pulp in spleens from LPS-challenged mice, and again, after combined treatment with AMD3100 and LPS, this effect was more pronounced.

**Fig. 8:**
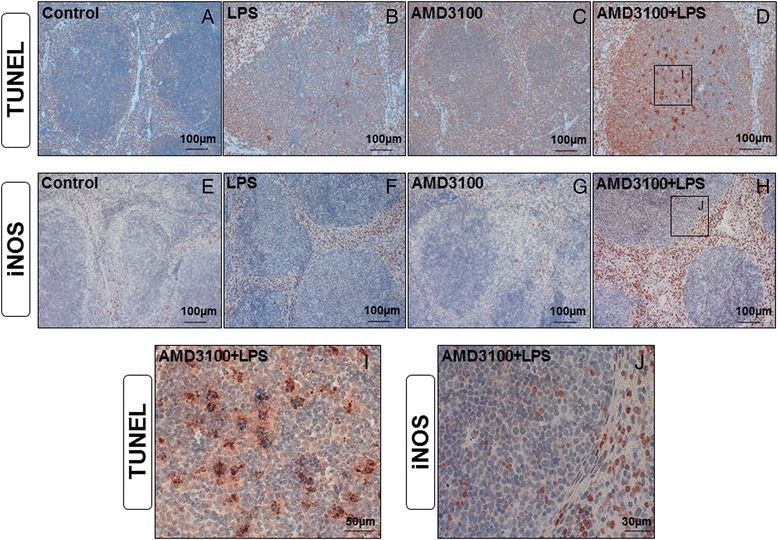
TUNEL assay and iNOS expression in the spleens. C57BL/6 N mice were treated either with LPS (5 mg/kg body weight), AMD3100 (5 mg/kg body weight), with both substances or with the solvent PBS (control). 24 h thereafter, the mice were sacrificed and the spleens were collected for immunohistological analysis (red-brown color, counterstaining with hematoxylin). Representative photomicrographs from one of seven different tissue samples stained by means of the TUNEL assay or for iNOS expression are shown (original magnification: (**a**-**h**) 200×, (**i**, **j**) 630×)

Because apoptotic processes are of substantial importance in endotoxemia, we subsequently determined the amount, and the distribution of cells undergoing apoptosis using TUNEL staining (Figs. 8a-d, and i). We found that 24 h post-treatment, endotoxin caused an elevated apoptosis in the white pulp. In particular, tingible body macrophages appeared to be the main locus of cell death, and co-treatment with AMD3100 further exacerbated these effects. By assessing the expression of cleaved caspase-3 with immunohistochemistry and immunoblotting (Fig. 7b), we were able to confirm these observations, as the CXCR4 blockade amplified the appearance of this pro-apoptotic enzyme. Finally, when determining iNOS expression, we detected several neutrophil granulocytes in both the red and the white pulp of co-treated mice (Figs. 2g-h and 8e-h, and j). Critically, although LPS alone enhanced the amount of iNOS-positive granulocytes in the red pulp, in comparison to control treatment, the additional CXCR4 blockade with AMD3100 amplified this effect in the red pulp and induced additional iNOS expression in the white pulp. Treatment with AMD3100 alone had no effect on iNOS expression in the spleen.

## Discussion

In contrast to other studies, which focused mainly on the general outcome of the animals and on the migration of several inflammatory cell populations in different inflammation models, we were primarily interested in the precise effects of CXCR4 blockade in endotoxemia. In particular, we focused on the systemic impact, as well as the influence on organ function. Furthermore, we intended to confirm previous investigations, which utilized different CXCR4 agonists [15, 16, 32], and in particular, our preceding study, in which we found that the CXCL12 analog CTCE-0214D exerted protective effects. Also, we aimed to indirectly show that CTCE-0214D signals through CXCR4 and to further unravel whether an interruption of the CXCR4/CXCL12 axis results in a deleterious outcome in endotoxemia.

Here, our findings demonstrate a devastating impact of AMD3100 in endotoxemia. This is consistent with our previous study, in which almost all parameters were improved after administration of the CXCL12 analog CTCE-0214D [14]. From these results, we concluded that this analog exerts its effects through CXCR4, and thus, CXCR4 exhibits a massive impact on systemic inflammation. This is also in good agreement with other investigations showing that CTCE-0214D and ubiquitin increase survival and diminish pro-inflammatory cytokine levels in vivo. Also, in line with our results, administration of AMD3100 in combination with LPS was associated with an increased mortality in zebrafish [33] and specific CXCL12 inhibitors revealed to be deleterious during sepsis induced by cecal ligation and puncture (CLP) [34].

CXCL12 and its receptor are mandatory for the migration of several cells, including monocytes, neutrophils, and hematopoietic progenitor cells. Delano et al. showed that mobilization of neutrophils to the sites of infection is CXCR4/CXCL12-dependent and that an interruption of the CXCR4/CXCL12 axis impaired bacterial clearance [34]. In addition, Guan et al. demonstrated that the CXCL12 analog, CTCE-0214D, recruits polymorphonuclear leukocytes (PMN) to the site of infection and enhances their phagocytic activity, thereby improving bacterial clearance in CLP-induced sepsis [32]. AMD3100 was also shown to promote inflammatory cell egress, simultaneously inhibiting their recruitment to sites of injury [35]. In addition, AMD3100 appeared to cause a preferential release of neutrophils from the lungs, while inhibiting their return to the bone marrow for elimination from the blood [36], indicating that CXCR4 blockade can negatively influence physiological defense mechanisms. These findings may serve as possible explanations for our observations, as we detected a larger number of neutrophil granulocytes in the livers and spleens of mice treated with AMD3100 plus LPS, in comparison to LPS-only treated animals. This suggests that neutrophils entered the tissues and their return to the bone marrow was likely blocked by the interrupted CXCR4/CXCL12 signaling. This hypothesis is also supported by the severe apoptosis observed in the white pulp of the spleen, as the large (unnecessary and nonfunctional) cell amount is probably answered by an increased cell death.

Furthermore, we identified fewer CD3+ cells in the spleens, but evidently more CD3+ cells in the livers of AMD3100 plus LPS co-treated mice, as compared to the LPS-only treated controls, suggesting that CXCR4 blockade “shifts” pro-inflammatory cells to the livers to provoke an inflammatory condition. Investigations by Tsuchiya et al. revealed CXCR4 conditional knock-out mice to exhibit increased numbers of CD3+ cells in the liver when inducing a chronic liver disease with CCl4 [37], whereas others were not able to detect such signs of inflammation in the liver when blocking CXCR4 [35]. Besides, we detected a reduced number of lymphocytes in the bloodstream after 24 h, suggesting severe disease progression, which seems to be a critical challenge for the immune system. An enhanced degree of apoptosis, mainly in the thymus and spleen, as well as the intensified immigration of lymphocytes into the tissues, may serve as possible explanations. This also applies to the neutrophil count in the blood. As we observed more neutrophils in the organs, we hypothesize that CXCR4 blockade promotes their migration from the bloodstream. This is supported by the observation that LPS and AMD3100 alone induce neutrophilia when compared to the control mice. However, these results warrant further investigations. Altogether, we assume that migration plays a predominant role in the observed phenotypes, as more CD68+ and CD8+ cells were found in the spleens of co-treated mice, suggesting macrophages and cytotoxic T cells to immigrate after AMD3100 administration in endotoxemia.

It is important to note that the complexity of the CXCR4/CXCL12 axis has previously been clearly demonstrated. For example, a study found that both a three-fold increase in neutrophils, known to be injurious, and a two-fold increase in hematopoietic stem cells, known to be protective, occurred simultaneously at 36 h post-injury. Here, the authors hypothesized that the neutrophilic predominance may have mitigated any potential beneficial effects from the hematopoietic stem cells [35, 38]. These findings may help to explain the seemingly contradictory results obtained in different studies, such as a beneficial impact of a CXCR4 blockade in autoimmune diseases and a deleterious influence in ischemia and acute inflammation [14, 39].

In accordance with previous investigations, which found that CXCR4 agonists reduce serum levels of pro-inflammatory cytokines in endotoxemia [14–16], we detected increased levels of serum TNF alpha and IFN gamma after CXCR4 blockade. Also Bach et al. detected increased TNF alpha levels in polytrauma after AMD3100 and LPS challenge [40]; however, the exact cause of these observations remains unknown. As previously stated, CXCR4 may influence TLR4 signaling and consequently lead to increased inflammatory responses. However, whether an activation or blockade is beneficial remains unclear and seems to depend on the cell line, the in vitro stimulus, and on the type of inflammation in vivo [19, 41]. As in other experiments, we attempted to elucidate if THP-1 monocytes and macrophages are affected after challenge with LPS alone, or in combination with AMD3100 or CXCL12. However, we were unable to detect any impact on TNF alpha secretion (Additional file 1 d, e). We therefore hypothesize that a direct role for CXCR4 in TLR4 signaling is secondary, and that migration likely plays the predominant role. The infiltrating neutrophils and T lymphocytes may have contributed to the observed deleterious conditions, as they are able to produce large amounts of pro-inflammatory cytokines. However, we discovered that, besides the infiltrating cells, endothelial cells in the liver are the major source of TNF alpha production. Whether these cells are directly activated by LPS, or are stimulated by a previous activation, e.g., from Kupffer cells or neutrophils, warrants further investigation. Up until now, there have been few studies focusing on endothelial cells as a source of pro-inflammatory cytokine production. In one case, it was found that HUVECs stimulated with IL-1 alpha produced a moderate amount of TNF alpha, whereas LPS did not induce TNF alpha secretion [42]. This underscores the complexity of the CXCR4/CXCL12 axis in inflammatory diseases.

We further found that AMD3100 in combination with endotoxin caused massive oxidative stress, particularly in the brains, kidneys, and livers, which can lead to a severe damage of several cell structures, including lipids and membranes, proteins, and DNA [43]. As mentioned, we detected several neutrophils in the livers of co-treated mice that likely contributed to the increased TNF alpha and IFN gamma production. Both cytokines have been reported to generate ROS via mitochondria and NADPH oxidase and are able to heavily upregulate iNOS [44, 45]. We confirmed these observations in our study, as we identified several neutrophils in the livers of the co-treated mice expressing iNOS. Correspondingly, we measured elevated serum NO levels that may have contributed to the massive oxidative stress in all organs investigated. Furthermore, NO is known to be involved in hypotension, cardiodepression, and vascular hyporeactivity in septic shock [46]. Therefore, it may have negatively influenced the general well-being of the mice, as evidenced by their decreased health status.

Interestingly, immunoblot and immunohistochemistry analysis further revealed reduced HO-1 levels after AMD3100 challenge in endotoxemia. The protective effects of HO-1 have been clearly demonstrated, as HO-1-deficient mice were found to be more susceptible to microbial sepsis [47]. HO-1 functions as an anti-oxidative and anti-inflammatory enzyme, and several authors have reported decreased TNF alpha and ROS levels after its activation [48]. In endotoxemia, oxidative stress plays a critical role, and attenuation of a central enzyme, such as HO-1, is likely to have detrimental effects. Here, our findings are in agreement with our previous study, which found that the CXCL12 analog CTCE-0214D induced the HO-1 expression and activity in the liver [14]. Furthermore, co-treatment with AMD3100 and LPS further diminished the levels of Nrf-2 in the liver, suggesting CXCR4 blockade to be pro-oxidative, as activation of Nrf-2 results in the induction of many cytoprotective proteins, such as NAD(P)H quinone oxidoreductase 1, glutamate-cysteine ligase, and HO-1 [49].

As we further measured a decreased biotransformation capacity in livers from mice treated with AMD3100 plus LPS, as compared to the LPS-only group, their ability to detoxify and eliminate LPS-induced secondary products, such as reactive oxygen species, was even more restricted. Consequently, the fewer CYP families are active, the fewer proteins are protected from degradation. Furthermore, biotransformation capacity is of critical importance, as septic patients are frequently treated with several drugs, such as antibiotics. Therefore, we postulate that CXCR4 blockade could further impair a patient’s overall outcome by enhancing the side effects from multiple medications. Again, with AMD3100, we detected results that are consistent with our previous study, in which we showed that CTCE-0214D treatment improved the biotransformation capacity and the oxidative status in the liver in endotoxemia. Together, these results suggest that modulation of CXCR4 is of critical importance in this disease.

In addition to biotransformation capacity, treatment with AMD3100 in conjunction with LPS induced liver damage by causing fat accumulation and slight edema, along with increased serum ALAT and ASAT levels. These findings are in good agreement with a recent study, in which AMD3100 was used in combination with CCl4 [35]. Here, the mice showed a trend towards increased liver necrosis at 36 h, while ALAT and ASAT levels were slightly, but not significantly, enhanced. From these results, the authors concluded that there may be a trend towards increased hepatic inflammation when administering AMD3100 in combination with CCl4. Furthermore, the decreased liver glycogen content and the reduced blood glucose levels indicate a massive glucose consumption. Kupffer and endothelial cells are the major producers of various prostaglandins, which have been shown to trigger glycogenolysis in the liver [50, 51]. In addition, TNF alpha decreased gluconeogenesis in hepatocytes isolated from 10-day-old rats [52], suggesting an excessive activation of inflammatory cells to be responsible for the alteration of the glucose homeostasis.

In order to further determine the impact of CXCR4 blockade on the kidneys, we measured the concentrations of urea and creatinine in the serum (Additional file 1 b, c). We postulate that the reduced serum urea levels seen after LPS treatment, and that become more pronounced after co-treatment with AMD3100, are attributable to impaired liver function, as the liver produces the majority of urea. Only in cases where kidney function is limited to 30 % or less, do serum urea levels increase, and thus, this parameter may serve as a measure for kidney damage. Consequently, our data also suggest that liver function is more severely affected than kidney function in endotoxemia. However, after AMD3100 plus LPS administration, serum creatinine was significantly enhanced, suggesting that kidney function is slightly impaired, at least in this instance.

As reported, co-administration of AMD3100 and LPS caused splenomegaly, mainly due to an increased number of erythrocytes and neutrophils in the red pulp and a slight edema, as well as the occurrence of some neutrophils in the white pulp. Splenomegaly is a well-known response to LPS challenge, in which nonfunctional erythrocytes and white blood cells are degraded. Apparently, CXCR4 blockade resulted in more cells in need of elimination that subsequently entered the spleen tissue, resulting in a more severe inflammation and an impaired health status. Correspondingly, we observed massive apoptosis in the white pulp after co-administration of AMD3100 and LPS. Further, cleaved caspase-3 expression was strongly increased, confirming a role for apoptosis. These observations are in accordance with our previous investigations, in which the CXCL12 analog was able to mitigate apoptosis in the white pulp, once again suggesting CXCR4 modulation is of critical importance in endotoxemia.

## Conclusions

In summary, in the present investigation, we demonstrated that CXCR4 blockade with AMD3100 exerts deleterious effects in multiple facets of endotoxemia. Specifically, we found that AMD3100 treatment in conjunction with LPS further worsened the health status, augmented pro-inflammatory cytokine expression, increased oxidative stress, and induced apoptosis in different organs, as compared to LPS alone. Further, this blockade mediated an additional decrease in liver biotransformation capacity, as well as a reduction in HO-1 expression. We also observed an increased immigration of several inflammatory cell populations into the tissues in mice co-treated with AMD3100 plus LPS, which likely contributed to the devastating overall outcome observed in these animals. The present results are consistent with previous findings, in which different CXCR4 agonists were found to exert beneficial effects in endotoxemia in vivo. In addition, by detecting effects with the CXCR4 antagonist, AMD3100, that are in direct contrast to those observed with the CXCL12 analog, CTCE-0214D, in endotoxemia, we can hypothesize that treatment with CTCE-0214D exerts its protective effects mainly via CXCR4. Collectively, these data enhance our understanding of the CXCR4/CXCL12 axis in inflammatory diseases and help to clarify the rare, and sometimes contradictory studies, in this field.

## Additional file

Additional file 1: Protein content in the liver, serum creatinine and urea levels and TNF alpha assay in THP-1 monocytes as well as macrophages. C57BL/6 N mice were treated either with LPS (5 mg/kg body weight), AMD3100 (5 mg/kg body weight), with both substances or with the solvent PBS (control). 24 h thereafter, the mice were sacrificed and the livers were collected for the determination of the protein content in the 9000 g supernatants by using the Biuret method (A). Also, the blood was obtained and serum creatinine as well as serum urea levels were measured (B, C). Data are given as mean ± standard error of the mean (SEM); *n* = 7 for each group. Statistical significant differences between the different treatment groups were determined by using the one-way analysis of variance (ANOVA) and the Tukey post hoc test and are indicated as follows: *, *p* < 0.05; **, *p* < 0.01; ***, *p* < 0.001 vs. control animals; ^+^, *p* < 0.05; ^++^, *p* < 0.01; ^+++^, *p* < 0.001 vs. LPS treatment. THP-1 cells were cultured in RPMI 1640 supplemented with 10 % fetal bovine serum and 100 nM penicillin/streptomycin (all Capricorn Scientific, Ebsdorfergrund, Germany) and were cultured at a density of about 1.0 × 10^6^/ml at 5 % CO_2_ at 37 °C. To obtain THP-1 macrophages, cells were differentiated with PMA (phorbol 12-myristate 13-acetate; Sigma-Aldrich, St. Louis, MO) at a concentration of 40 ng/ml for three days. For the experiments, the cells were pretreated with AMD3100 or CXCL12, respectively, 30 min before LPS was added. After additional 12 h, the media were collected and analyzed for TNF alpha concentration by using a TNF alpha ELISA Kit (Thermo Fisher Scientific, Massachusetts, USA) (D, E). Data are given as mean ± standard error of the mean (SEM); *n* = 3 independent experiments. (PDF 169 kb)

## Abbreviations

AP-1: Activator protein 1
CD3/6/8/14/55/68: Cluster of differentiation 3/6/8/14/55/68
CLP: Cecal ligation and puncture
CXCL12: CXC motif chemokine 12
CXCR4: CXC chemokine receptor type 4
CYP: Cytochrome P450
GSH: Reduced glutathione
GSSG: Oxidized glutathione
HEK cells: Human embryonic kidney cells
HO-1: Heme oxygenase 1
HUVEC: Human umbilical vein endothelial cell
IFN gamma: Interferon gamma
IgG: Immunoglobulin G
IL: Interleukin
LPO: Lipid peroxides
LPS: Lipopolysaccharides
MD-2: Myeloid differentiation protein-2
MIF: Macrophage migration inhibitory factor
NF-kB: Nuclear factor ‘kappa-light-chain-enhancer’ of activated B-cells
NO: Nitric oxide
Nrf-2: Nuclear factor (erythroid-derived 2)-like 2
PBS: phosphate-buffered saline
PI3K: Phosphatidylinositol-4,5-bisphosphate 3-kinase
PVDF: Polyvinylidene fluoride
ROS: Reactive oxygen species
SDS: Sodium dodecyl sulfate
TLR4: Toll-like receptor 4
TNF alpha: Tumor necrosis factor alpha
TUNEL: TdT-mediated dUTP-biotin nick end labeling
VEGF: Vascular endothelial growth factor
