# Supplementary material for: Administration of AMD3100 in endotoxemia is associated with pro-inflammatory, pro-oxidative, and pro-apoptotic effects in vivo
Source: J Biomed Sci. 2016 Oct 3;23:68. doi: 10.1186/s12929-016-0286-8 (PMC5048674; doi:10.1186/s12929-016-0286-8)
Supplement: Additional file 1: — Protein content in the liver, serum creatinine and urea levels and TNF alpha assay in THP-1 monocytes as well as macrophages. C57BL/6 N mice were treated either with LPS (5 mg/kg body weight), AMD3100 (5 mg/kg body weight), with both substances or with the solvent PBS (control). 24 h thereafter, the mice were sacrificed and the livers were collected for the determination of the protein content in the 9000 g supernatants by using the Biuret method (A). Also, the blood was obtained and serum creatinine as well as serum urea levels were measured (B, C). Data are given as mean ± standard error of the mean (SEM); n = 7 for each group. Statistical significant differences between the different treatment groups were determined by using the one-way analysis of variance (ANOVA) and the Tukey post hoc test and are indicated as follows: *, p < 0.05; **, p < 0.01; ***, p < 0.001 vs. control animals; +, p < 0.05; ++, p < 0.01; +++, p < 0.001 vs. LPS treatment. THP-1 cells were cultured in RPMI 1640 supplemented with 10 % fetal bovine serum and 100 nM penicillin/streptomycin (all Capricorn Scientific, Ebsdorfergrund, Germany) and were cultured at a density of about 1.0 × 10^6^/ml at 5 % CO2 at 37 °C. To obtain THP-1 macrophages, cells were differentiated with PMA (phorbol 12-myristate 13-acetate; Sigma-Aldrich, St. Louis, MO) at a concentration of 40 ng/ml for three days. For the experiments, the cells were pretreated with AMD3100 or CXCL12, respectively, 30 min before LPS was added. After additional 12 h, the media were collected and analyzed for TNF alpha concentration by using a TNF alpha ELISA Kit (Thermo Fisher Scientific, Massachusetts, USA) (D, E). Data are given as mean ± standard error of the mean (SEM); n = 3 independent experiments. (PDF 169 kb) [file 12929_2016_286_MOESM1_ESM.pdf]

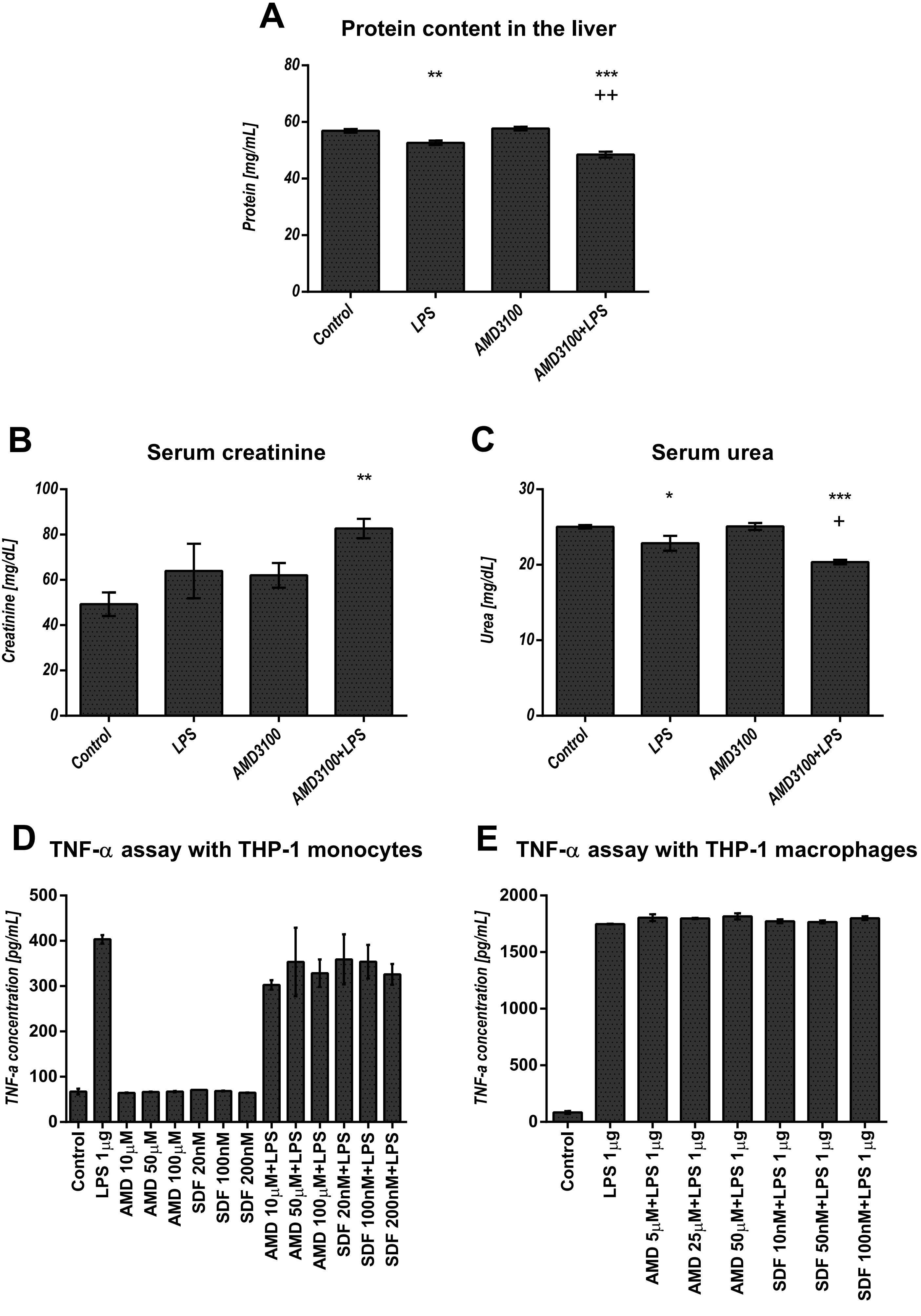

A
Protein content in the liver
Protein [mg/mL]
80
60
40
20
0
**
***
++
Control
LPS
AMD3100
AMD3100+LPS
B
Serum creatinine
Creatinine [mg/dL]
100
80
60
40
20
0
**
Control
LPS
AMD3100
AMD3100+LPS
C
Serum urea
Urea [mg/dL]
30
20
10
0
*
***
+
Control
LPS
AMD3100
AMD3100+LPS
D
TNF-α assay with THP-1 monocytes
TNF-a concentration [pg/mL]
500
400
300
200
100
0
Control
LPS 1μg
AMD 10μM
AMD 50μM
AMD 100μM
SDF 20nM
SDF 100nM
SDF 200nM
AMD 10μM+LPS
AMD 50μM+LPS
AMD 100μM+LPS
SDF 20nM+LPS
SDF 100nM+LPS
SDF 200nM+LPS
E
TNF-α assay with THP-1 macrophages
TNF-a concentration [pg/mL]
2000
1500
1000
500
0
Control
LPS 1μg
AMD 5μM+LPS 1μg
AMD 25μM+LPS 1μg
AMD 50μM+LPS 1μg
SDF 10nM+LPS 1μg
SDF 50nM+LPS 1μg
SDF 100nM+LPS 1μg
